# Metabolic signatures of combined exercise and fasting: an expanded perspective on previous telomere length findings

**DOI:** 10.3389/fragi.2024.1494095

**Published:** 2024-11-20

**Authors:** Shamma Almuraikhy, Khaled Naja, Najeha Anwardeen, Maha Sellami, Hadaia Saleh Al-Amri, Haya Al-Sulaiti, Sara S. Bashraheel, Amina Ali Aden, Mohamed A. Elrayess

**Affiliations:** ^1^ Biomedical Research Centre, Qatar University, Doha, Qatar; ^2^ Sport Coaching Department, College of Sport Sciences, Qatar University, Doha, Qatar; ^3^ Department of Biomedical Sciences, College of Health Sciences, QU Health, Qatar University, Doha, Qatar; ^4^ College of Medicine, QU Health, Qatar University (QU), Doha, Qatar; ^5^ Heart hospital, Out-patient Department, Hamad Medical Corporation (HMC), Doha, Qatar

**Keywords:** aging, telomere length, metabolomics, fasting, exercise

## Abstract

**Introduction:**

Aging is a complex process marked by a gradual decline in physiological function and increased susceptibility to diseases. Telomere length is frequently regarded as one of the primary biomarkers of aging. Metabolic profiles are key features in longevity and have been associated with both age and age-related diseases. We previously reported an increase in the telomere length in healthy female subjects when Ramadan fasting was combined with physical training. This study aims to characterize the metabolic signature differentiating the combined effects of exercise and fasting from exercise alone and explore the correlations with the previously reported telomere length changes.

**Methods:**

Twenty-nine young, non-obese, and healthy female subjects were previously randomized into two groups: one group followed a 4-week exercise program, while the other group followed the same 4-week exercise program but also fasted during Ramadan. Metabolic profiles were assessed pre- and post-intervention using untargeted metabolomics.

**Results and Discussion:**

Our results showed a significant decrease in many lipid metabolites in the exercise-while-fasting group, particularly ceramides. Our study sheds light on the dynamic changes in lipid metabolism and its potential role in inflammation and age-related diseases, and contributes to the broader understanding of how lifestyle factors can influence cellular aging and metabolic health.

## 1 Introduction

In recent decades, research in aging has gained significant traction, driven by the understanding that aging is not merely a passive biological process but can be influenced by genetic and lifestyle factors. Telomere length is often considered one of the key biomarkers of aging. Metabolomics is a powerful tool that holds great significance in advancing our understanding of aging. It enables researchers to examine the influence of external factors, such as diet and physical activity on the aging process (Panyard et al., 2022). Notably, changes at the metabolic level are key features in longevity, and metabolic profiles have been associated with age and age-related diseases (van der Spek et al., 2019). Recent studies have developed novel metabolomic aging scores that predict mortality and disease risk more accurately than traditional metrics (Zhang et al., 2024). An increasing amount of research indicates that lipid metabolism plays a significant role in the aging process (Johnson and Stolzing, 2019; Pablo Palavicini and Han, 2021). Understanding these metabolic changes can help identify potential interventions and lifestyle modification aimed at enhancing health span.

Both exercise and intermittent fasting play vital roles in shaping the human metabolome through distinct yet complementary mechanisms. Exercise induces acute and chronic changes in metabolite concentrations, affecting energy metabolism, lipid metabolism, and amino acid metabolism (Pellegrino et al., 2022; Jaguri et al., 2023). On the other hand, intermittent fasting alters metabolic pathways by promoting fat oxidation and enhancing ketogenesis, which shifts the body’s energy source from carbohydrates to fats (Washburn et al., 2019; Madkour M. et al., 2023). The combination of exercise and intermittent fasting can yield synergistic effects, resulting in better metabolic health outcomes (Real-Hohn et al., 2018).

We previously reported (Almuraikhy et al., 2024), in a longitudinal study, that combining exercise with Ramadan fasting may be an effective tool for slowing down the aging process. In the exercise-while-fasting group, we observed a significant increase in the telomere length compared to the control group, which engaged in exercise alone and showed no notable change. Furthermore, our findings suggested that TNF-α and HDL may contribute to the observed variations in the telomere length, due to their opposing effects on inflammation and oxidative stress. The objective of this study is to use untargeted metabolomics analysis to characterize the metabolic signature that differentiated between the combined effects of exercise and fasting compared to the effect of exercise intervention only. By analyzing metabolites, we hope to uncover specific biochemical pathways influenced by this dual intervention that may not be evident through traditional clinical measures alone. The findings of this study will contribute to the growing body of knowledge on telomere biology and its interplay with metabolic factors.

## 2 Methods

### 2.1 Study participants

A total of 29 healthy female subjects aged between 20 and 30 years, categorized as lean or overweight (with a BMI ranging from 20 to less than 30), were selected from Qatar University students for this study. The research received approval from the Qatar University Institutional Review Board (QU-IRB 1798-EA/23). All participants gave their informed consent and underwent an initial medical assessment to identify any potential health issues and confirm their eligibility for the training program. The participants were divided into two randomized groups: the first group (n = 16), considered as the control group (4W), completed a 4-week exercise training program. The second group (4W + F), consisting of 13 participants, underwent a 4-week exercise training program while fasting for 14 h during Ramadan. Ramadan fasting is a form of intermittent fasting. This practice involves a daily fast that lasts from dawn until dusk, during which participants abstain from food, drink, and smoking. The training program, adhering to the American College of Sports Medicine (ACSM) and American Heart association (AHA) recommendations (Samjoo et al., 2013; Marson et al., 2016; Battista et al., 2021; Kanaley et al., 2022), comprised aerobic exercises with progressive intensity. Exercise timing for both groups was in the afternoon period (1 to 3 p.m.). Anthropometric measurements and fasting blood samples were taken before and after intervention. Blood samples were collected by a licensed nurse between 8:00 a.m. and 12:00 p.m. during fasting. All samples were kept on ice, and the serum was separated within 1 hour after collection by centrifuging the blood for 15 min at 2500 rpm. For fasting blood glucose and lipid profile tests, the analyses were run immediately, and the remaining serum was aliquoted and stored at −80°C. Supplementary Table S1 provides an overview of participants in this study, grouped into (4W) and (4W + F), before and after intervention, respectively.

### 2.2 Metabolomics

Metabolomic profiling of serum samples from all participants was performed using Metabolon’s platform, according to standardized protocols (Al-Khelaifi et al., 2018). The Waters ACQUITY ultra-performance liquid chromatography (UPLC) instrument (Waters Corporation, Milford, MA, USA) coupled to a Thermo Scientific Q-Exactive high resolution/accurate mass spectrometer (Thermo Fisher Scientific, Waltham, MA, USA) interfaced with a heated electrospray ionization (HESI-II) source with the Orbitrap mass analyzer operated at 35,000 mass resolution. Details of the liquid chromatography–mass spectrometry (LC–MS) techniques utilized in this study were previously described (Al-Khelaifi et al., 2018; Al-Sulaiti et al., 2024). Compounds were identified by comparing them to library entries of purified standards or recurrent unknown entities, using a database of over 3,300 commercially available purified standard compounds. Library matches for each compound were verified for each sample and corrected when necessary (Evans, 2014; Al-Sulaiti et al., 2019).

### 2.3 Statistical analysis

Metabolomics data on 1039 known and 259 unknown identities were batch-normalized (median-scaled) before imputation was performed for missing values using a minimum value across batches from the median-scaled data. The data were natural log-transformed prior to statistical analysis. Unknown metabolites were excluded from the downstream statistical steps. Principal component analysis (PCA) was performed to assess whether there is a significant difference between the before- and after-treatment groups (Supplementary Figure S1). The highest discriminant metabolites between the intervention groups were found using an OPLS-DA model. Univariate analysis was conducted using fold change and paired Student’s t-test. The *p*-values were adjusted using false discovery rate (FDR) correction. Functional enrichment analysis was performed on the FDR significant metabolites list from the univariate analysis using Fisher’s exact test and was followed by the FDR multiple testing correction method. The sub-pathways were previously predefined using Metabolon, and those with less than three top hits were dropped. The Δ(T/S) ratio, which captures the change in the telomere length before and after exercise, was previously calculated for the same subjects (Almuraikhy et al., 2024). A partial correlation network was constructed using a Gaussian graphical model (GGM) to explore the relationships between the significant metabolites before and after exercise and the Δ(T/S) ratio in the 4WF group. The network was based on significant correlations (*p* < 0.05).

## 3 Results

### 3.1 Multivariate analysis of metabolites differentiating (4W) and (4W + F) before and after intervention

**FIGURE 1:**
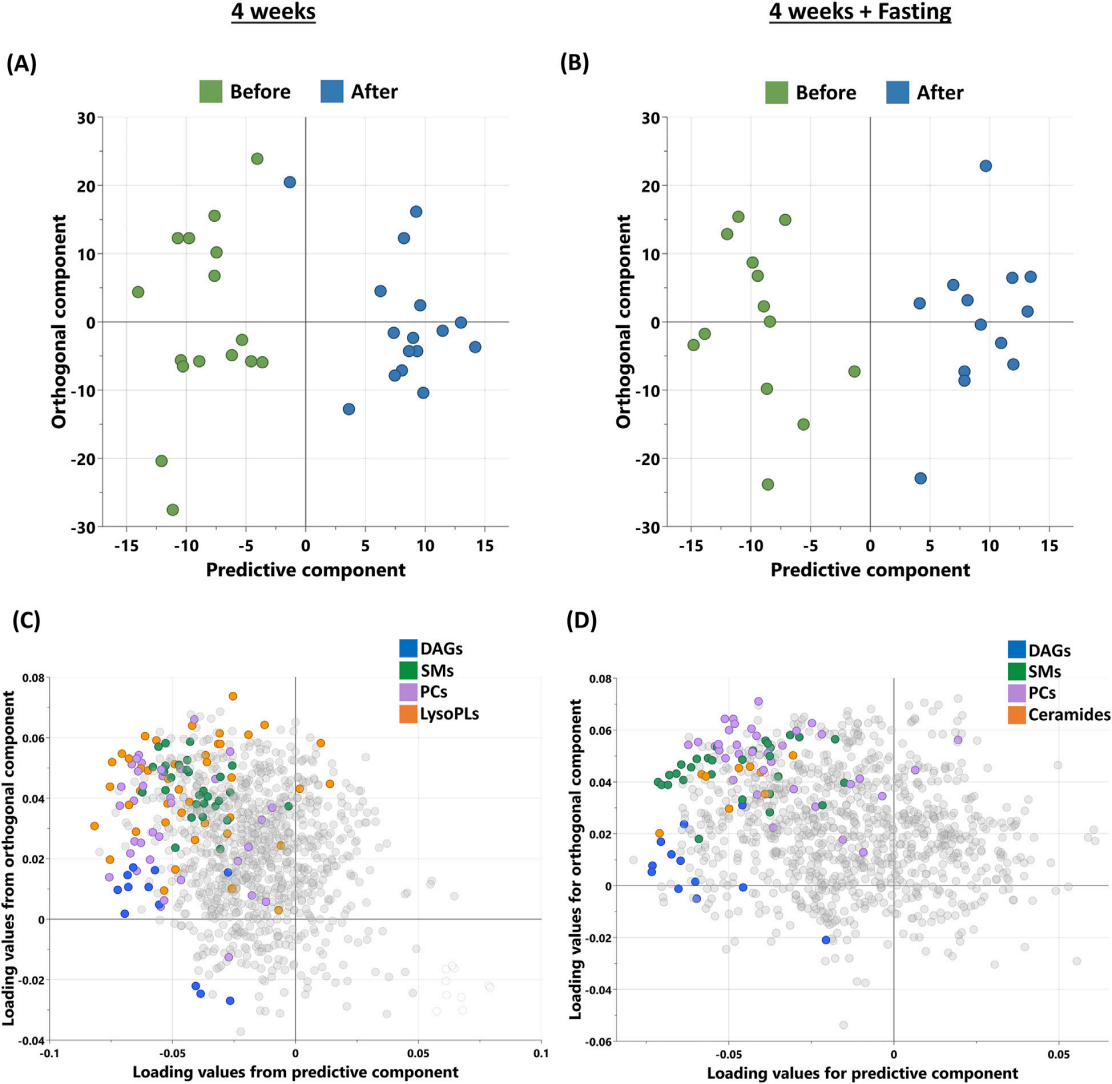
Metabolomics analysis of participants’ sera after 4 weeks of moderate physical training (n = 16) and 4 weeks training combined with fasting (n = 13). **(A, B)** OPLS-DA score plot of the participants before and after physical training in 4W (R^2^Y – 0.87, Q^2^ – 0.056) and 4W + F (R^2^Y – 0.895, Q^2^ – 0.331) groups, respectively. **(C, D)** Loading plots depicting the metabolites from significantly enriched pathways of the same. [DAGs, diacylglycerols; SMs, sphingomyelins; PCs, phosphatidylcholines; LysoPLs, lysophospholipids].

Non-targeted metabolomics analysis was used to identify the metabolic signatures of participants in this study, grouped into (4W) and (4W + F) before and after intervention. OPLS-DA was used to identify the best distinguishing components before and after, as shown in Figures 1A, B. OPLS-DA revealed one predictive and two orthogonal components, with the discriminatory component accounting for 87% of the variance between before and after intervention in the (4W) group and 89.5% of the variance in the (4W + F) group. The loading plot (Figures 1C, D) displays metabolites responsible for differentiation, including diacylglycerols (DAGs), sphingomyelins (SMs), and phosphatidylcholines (PCs), which are common between both groups and ceramides only in the (4W + F) group.

### 3.2 Univariate analysis of metabolites differentiating between before and after 4 weeks training

Paired Student’s t-test revealed FDR (≤0.05) significant changes in the (4W) group before and after exercise, as indicated in Table 1. Results showed a decrease in diacylglycerols, lysophospholipids, and acylcarnitines in this group. Dot-plot boxplots of the top significant metabolites are depicted in Supplementary Figure S2. Volcano plots showing all the increased and decreased metabolites post-exercise are shown in Supplementary Figure S4A.

**TABLE 1:** Results from the paired Student’s t-test showing metabolites differentiating the exercise group before and after intervention. (*) indicates a compound that has not been officially confirmed based on a standard but that Metabolon is confident in its identity.

Metabolite	Sub-pathway	Super-pathway	Log_2_FC	VIP	*p*-value	FDR
Oleoyl-arachidonoyl-glycerol (18:1/20:4) (van der Spek et al., 2019) *	Diacylglycerol	Lipid	−1.272	1.971	3.37 × 10^−5^	0.004
1-Docosahexaenoyl-GPC (22:6) *	Lysophospholipid	Lipid	−0.7930	2.07146	1.01 × 10^−4^	0.007
Linoleoyl–linoleoyl–glycerol (18:2/18:2) (van der Spek et al., 2019) *	Diacylglycerol	Lipid	−1.043	1.816	3.67 × 10^−4^	0.015
Propionylcarnitine (C3)	Fatty acid metabolism (also BCAA metabolism)	Lipid	−1.231	1.674	5.51 × 10^−4^	0.017
Linoleoyl–arachidonoyl–glycerol (18:2/20:4) (van der Spek et al., 2019) *	Diacylglycerol	Lipid	−1.259	1.882	6.13 × 10^−4^	0.017
Lauroylcarnitine (C12)	Fatty acid metabolism (acyl carnitine, medium chain)	Lipid	−1.118	1.645	3.61 × 10^−3^	0.041
Linoleoyl–linolenoyl–glycerol (18:2/18:3) (van der Spek et al., 2019) *	Diacylglycerol	Lipid	−1.461	1.629	5.30 × 10^−3^	0.049

### 3.3 Univariate analysis of metabolites differentiating between before and after 4 weeks training while fasting

Paired Student’s t-test revealed FDR (≤0.05) significant changes in the (4W + F) group before and after exercise, as indicated in Table 2. Results showed a decrease in diacylglycerols, acylcarnitines, ceramides, and one kynurenine metabolite in this group. Dot-plot boxplots of the top significant metabolites are depicted in Supplementary Figure S3. Volcano plots showing all the increased and decreased metabolites post-exercise are shown in Supplementary Figure S4B.

**TABLE 2:** Results from the paired Student’s t-test showing metabolites differentiating the exercise-while-fasting group before and after intervention. (*) indicates a compound that has not been officially confirmed based on a standard but that metabolon is confident in its identity.

Metabolite	Sub-pathway	Super-pathway	Log_2_FC	VIP	*p*-value	FDR
Palmitoyl–linoleoyl–glycerol (16:0/18:2) (Panyard et al., 2022) *	Diacylglycerol	Lipid	−2.090	1.793	6.91 × 10^−5^	0.025
Lauroylcarnitine (C12)	Fatty acid metabolism (acyl carnitine, medium chain)	Lipid	−1.022	2.176	1.04 × 10^−5^	0.025
N-palmitoyl-sphingosine (d18:1/16:0)	Ceramides	Lipid	−0.497	1.761	1.53 × 10^−4^	0.025
N-palmitoyl-sphingadienine (d18:2/16:0) *	Ceramides	Lipid	−0.496	1.983	3.8 × 10^−4^	0.025
Ceramide (d18:2/24:1, d18:1/24:2) *	Ceramides	Lipid	−0.573	1.71	4.0 × 10^−4^	0.025
Oleoyl–linoleoyl–glycerol (18:1/18:2) (Panyard et al., 2022)	Diacylglycerol	Lipid	−1.524	2.019	4.78 × 10^−4^	0.025
N-stearoyl-sphingosine (d18:1/18:0) *	Ceramides	Lipid	−0.529	1.33	5.3 × 10^−4^	0.025
Oleoyl–linoleoyl–glycerol (18:1/18:2) (van der Spek et al., 2019)	Diacylglycerol	Lipid	−1.322	2.022	7.74 × 10^−4^	0.025
N-formylkynurenine	Tryptophan metabolism	Amino acid	−1.031	1.708	7.83 × 10^−4^	0.025
Octanoylcarnitine (C8)	Fatty acid metabolism (acyl carnitine, medium chain)	Lipid	−1.425	1.927	1.07 × 10^−3^	0.026
Decanoylcarnitine (C10)	Fatty acid metabolism (acyl carnitine, medium chain)	Lipid	−1.390	1.939	1.24 × 10^−3^	0.027
Oleoyl–arachidonoyl–glycerol (18:1/20:4) (van der Spek et al., 2019) *	Diacylglycerol	Lipid	−1.626	1.967	1.37 × 10^−3^	0.027
Linoleoyl–linoleoyl–glycerol (18:2/18:2) (van der Spek et al., 2019) *	Diacylglycerol	Lipid	−1.728	1.797	1.61 × 10^−3^	0.029
Myristoylcarnitine (C14:1) *	Fatty acid metabolism (acyl carnitine, monounsaturated)	Lipid	−1.518	1.736	1.91 × 10^−3^	0.031
Acetylcarnitine (C2)	Fatty acid metabolism (acyl carnitine, short chain)	Lipid	−2.776	1.692	2.42 × 10^−3^	0.035
Palmitoylcarnitine (C16:1) *	Fatty acid metabolism (acyl carnitine, monounsaturated)	Lipid	−1.016	1.581	3.32 × 10^−3^	0.044
1-Arachidonoyl-GPC (20:0)	Lysophospholipid	Lipid	−1.563	1.319	4.15 × 10^−3^	0.047
Linoleoyl–linoleoyl–glycerol (18:2/18:2) (Panyard et al., 2022) *	Diacylglycerol	Lipid	−1.724	1.646	4.35 × 10^−3^	0.048

### 3.4 Functional enrichment analysis of metabolites differentiating between before and after intervention in (4W) and (4W + F) groups

**FIGURE 2:**
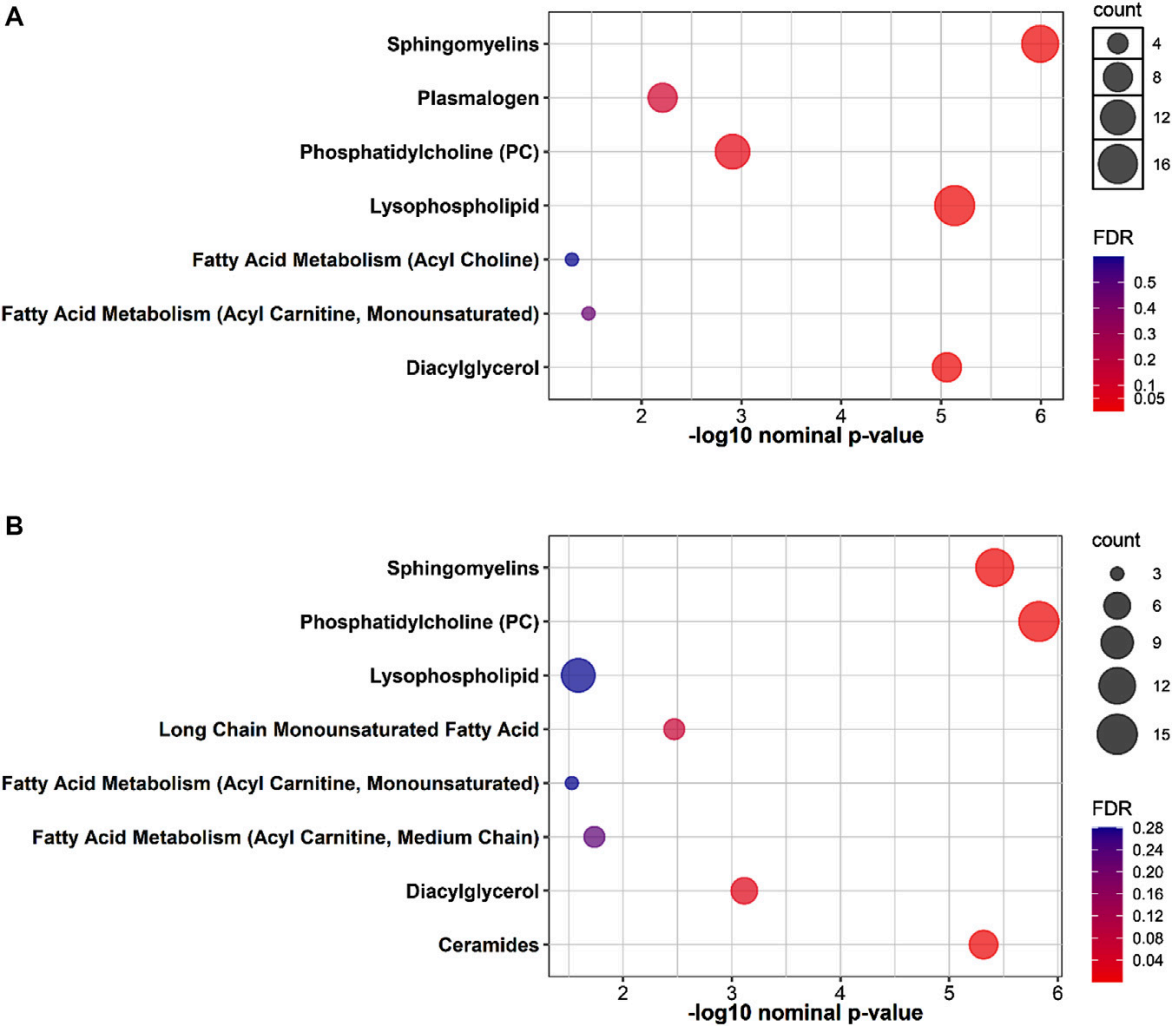
Plots showing the results of metabolite set enrichment analysis using Fisher’s exact test for **(A)**: 4W group and **(B)**: 4W + F group.

The results of functional enrichment analyses (Figure 2) indicated significant differences in sphingomyelins, phosphatidylcholines, lysophospholipids, diacylglycerol, acetylcholines, and acylcarnitines in both groups, whereas the exercise-while-fasting group showed a significant difference in ceramides.

### 3.5 Partial correlation network in the 4W + F group

**FIGURE 3:**
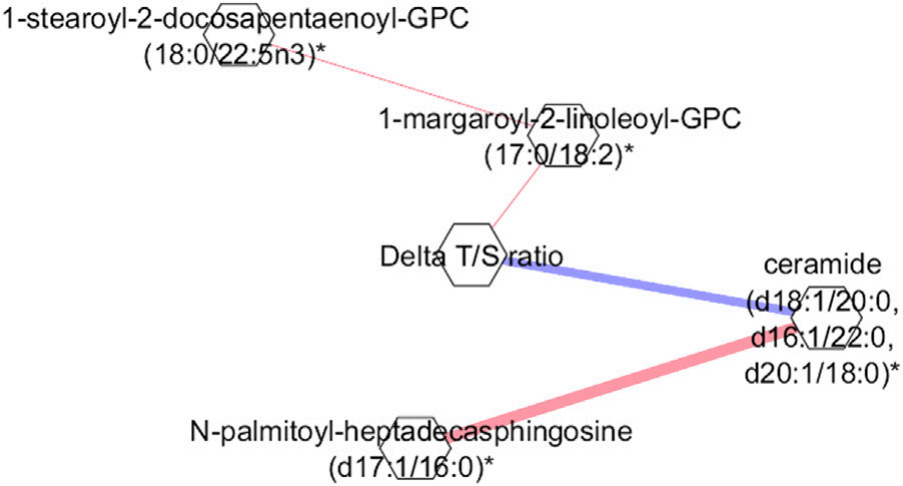
Gaussian graphical model (GGM) representing the partial correlations between the Δ(T/S) ratio and the metabolites in the 4W + F group. Red color indicates positive correlation. Blue color indicates negative correlation. Each node corresponds to a metabolite, and the edges indicate partial correlations, with the thickness of the edges reflecting the strength of these correlations.

To reveal key metabolic interactions influenced by exercise in the 4WF group, a partial correlation network was created using a GGM to investigate the relationships between the significant metabolites before and after exercise with the difference in the T/S ratio in the 4WF group. Figure 3 shows that the Δ(T/S) ratio is strongly and negatively correlated with ceramides.

## 4 Discussion

Changes at the metabolic level are crucial factors influencing longevity. Exercising while fasting may be an effective tool for slowing down the aging rate and contribute to overall wellbeing. The aim of this research is to identify and characterize the distinct metabolic signatures that emerged from the combined effects of exercise and fasting, in contrast to the effects of exercise alone. Analyzing these metabolites allows us to pinpoint the specific biochemical and metabolic changes induced by the combination of exercise and fasting. These unique metabolites may serve as potential biomarkers or targets for further investigations.

Our results showed a significant reduction in many ceramides and their derivatives in the exercise-while-fasting group only. Moreover, our GGM showed a strong negative correlation between the telomere length and ceramides. Ceramides are a type of sphingolipids that play a crucial role in regulating various cellular responses, such as senescence, apoptosis, and, more recently, autophagy (Hannun and Obeid, 2011). Kazmirczak et al. (2023) showed that intermittent fasting is effective in the attenuation of ceramide accumulation. However, Madkour et al. demonstrated a decrease in many sphingolipids, but not ceramides, upon Ramadan intermittent fasting (Madkour M. I. et al., 2023). The effects of exercise on ceramides are complex and depend on various factors such as the exercise duration, type, intensity, and the metabolic status of the individuals. Carrard et al. (2021) and Tan-Chen et al. (2020) reported in their reviews that while regular exercise is associated with a reduction in plasma ceramide levels, acute exercise may lead to a transient increase in the concentrations of these metabolites. However, Bergman et al. (2015) showed that chronic exercise training does not change plasma ceramide levels. This suggests that the notable reduction in ceramide levels observed in our study may result from the combined effects of exercise and fasting.

Interestingly, a close connection between ceramide levels and aging has been revealed (Mencarelli and Martinez–Martinez, 2013), and ceramides have been shown to be as one of the key upstream regulators of telomerase activity (Sundararaj et al., 2004a). Notably, C18-ceramide, generated by the enzyme ceramide synthase 1, has been found to downregulate the expression of telomerase (Wooten-Blanks et al., 2007). Relatedly, Laurila et al. reported an accumulation of ceramides in the skeletal muscle upon aging (Laurila et al., 2022). Moreover, treatment with exogenous ceramides was demonstrated to cause a rapid shortening of the telomere length (Sundararaj et al., 2004b) and trigger a senescent-like phenotype in young cells (Venable and Yin, 2009). Our previous research (Almuraikhy et al., 2024) indicated that TNF-α and HDL might play a role in the observed variations in the telomere length due to their contrasting impacts on inflammation and oxidative stress. Interestingly, ceramides were found to be inversely correlated with HDL (Meeusen et al., 2018), and TNF-α was demonstrated to induce and accelerate ceramide production (Hernández-Corbacho et al., 2015; Chaurasia et al., 2020). Reducing ceramide levels through exercise combined with fasting or blocking its effects on telomeres may be a promising anti-aging strategy. More research is needed to fully elucidate the mechanisms by which ceramides regulate telomere biology and influence the aging process.

Our results also showed a reduction in acylcarnitines and phosphatidylcholine in both groups; however, the reduction appears to be more pronounced in the exercise-while-fasting group. Recent studies suggest that aging may contribute to the generation of carnitine metabolites (Lo et al., 2023; Huguenard et al., 2023). Moreover, acylcarnitines and phosphatidylcholines were associated, among other metabolites, with a shorter telomere length (van der Spek et al., 2019; Subedi et al., 2023). Markedly, carnitine palmitoyltransferase 1C, the enzyme responsible for the formation of acylcarnitines, was shown to play a role in regulating cellular senescence and telomere length (Wang et al., 2020). Intriguingly, acylcarnitines have been reported to induce the secretion of TNF-α among other inflammatory cytokines (Enooku et al., 2019) and that the energy metabolism pattern of immune cells is determined by the availability of acylcarnitines, directing the activation of immune cell toward a pro-inflammatory phenotype (Dambrova et al., 2022; Rutkowsky et al., 2014). However, phosphatidylcholines are generally recognized for their anti-inflammatory properties (Treede et al., 2007). This paradoxical decrease in phosphatidylcholines could be related to their use as an energy source during fasting and exercise or may reflect alterations in membrane composition rather than just inflammatory status. This highlights the need for future comprehensive studies, which could provide a deeper understanding of the complex metabolic and inflammatory responses to fasted exercise and their potential relation with aging.

Our results showed a decrease in lysophospholipids and diacylglycerols in both groups, with a more marked decrease in the 4W group. Lysophospholipids are critical for maintaining mitochondrial membrane integrity and function, while diacylglycerols are crucial lipid molecules that play a significant role in cellular signaling and metabolism. Interestingly, research identified a significant downregulation of lysophospholipid levels in older adults (Pan et al., 2023). Additionally, overexpression of diacylglycerol lipase in model organisms has been linked to extended lifespans and enhanced resistance to oxidative stress (Lin et al., 2014). Our findings indicate that the 4W + F group exhibited a lesser impact on lysophospholipid and diacylglycerols levels compared to the 4W group. This observation suggests that fasting may confer a protective effect on the levels of these lipids.

The univariate analysis showed a significant decrease in N-formylkynurenine only in the exercise-while-fasting group. N-formylkynurenine is a metabolite in the kynurenine pathway, which is involved in the degradation of tryptophan. Strikingly, N-formylkynurenine was found to increase with age (Refaey et al., 2017). Moreover, TNF-α was shown to play a crucial role in directing tryptophan metabolism toward the kynurenine pathway, thereby increasing levels of kynurenine and its metabolites, including N-formylkynurenine (Okamoto et al., 2023). Despite these insights, there remains a notable gap in the literature concerning the relationship between N-formylkynurenine with both exercise and fasting, highlighting an important area for future research.

Although research indicates that the single-gene T/S ratio method yields reliable and reproducible results when executed correctly (Martin-Ruiz et al., 2015), we recognize that our study’s assessment of the telomere length was limited to the T/S ratio derived from a single reference gene. This represents a notable limitation in our research. It is essential to emphasize that the fasting duration is standardized for all participants throughout Ramadan. This intentional decision aims to maintain uniformity in fasting times and ensure compliance among everyone involved. Although participants were encouraged to follow a balanced diet, they were not required to adhere to a specific, predetermined dietary plan. This lack of a defined dietary regimen may be viewed as an additional limitation to our study, potentially influencing their metabolic profiles. The relatively short duration of this study is another limitation. A longer follow-up period would have allowed us to observe potential long-term changes in metabolite profiles and assess the stability of the observed effects over time. Additionally, it is important to acknowledge that the metabolite changes reported in this study were not confirmed or validated using a secondary analytical method. Future studies should consider employing complementary techniques. Moreover, it is crucial to recognize that, although we can speculate on the possible connections between the observed metabolic changes and variations in the telomere length, the existing evidence remains preliminary. Currently, it does not establish any causal relationship, and further research is necessary to deepen our understanding of this connection.

## 5 Conclusion

Our study demonstrates that the combination of exercise and fasting leads to significant metabolic alterations, particularly a notable reduction in ceramide levels, which may play a crucial role in promoting longevity and overall health. This synergistic approach not only affects ceramide metabolism but also influences other key metabolites, such as acylcarnitines, lysophospholipids, diacylglycerol, and phosphatidylcholine, underscoring the relationship between metabolism, inflammation, and cellular vitality. By identifying specific metabolites as potential biomarkers, our research paves the way for targeted interventions that may further promote healthy aging and longevity. Future comprehensive studies could offer deeper insights into the complex metabolic and inflammatory responses to fasted exercise and their complex relationship with aging.

## Data Availability

The original contributions presented in the study are included in the article/Supplementary Material; further inquiries can be directed to the corresponding author.
